# Global, regional, and national trends in type 2 diabetes mellitus burden among adolescents and young adults aged 10–24 years from 1990 to 2021: a trend analysis from the Global Burden of Disease Study 2021

**DOI:** 10.1007/s12519-024-00861-8

**Published:** 2025-01-03

**Authors:** Si-Te Xu, Mu Sun, Yu Xiang

**Affiliations:** 1 Department of Statistics, Ruijin Hospital, Shanghai Jiaotong University School of Medicine, Shanghai, 200025 China; 2 Department of Endocrine and Metabolic Diseases, Shanghai Institute of Endocrine and Metabolic Diseases, Ruijin Hospital, Shanghai Jiaotong University School of Medicine, Shanghai, 200025 China

**Keywords:** Adolescent, Burden, GBD, T2DM, Trend analysis

## Abstract

**Background:**

Type 2 diabetes mellitus (T2DM) poses an escalating public health challenge among adolescents and young adults worldwide. Despite the rising incidence, comprehensive data on the burden and trends of T2DM in this demographic remain scarce. This study aims to evaluate the burden of T2DM among individuals aged 10–24 years globally, regionally, and nationally from 1990 to 2021.

**Methods:**

Utilizing data from the Global Burden of Diseases, Injuries, and Risk Factors Study (GBD) 2021, we assessed incidence rates, disability-adjusted life-years (DALYs), and average annual percentage changes (AAPCs) for T2DM in the specified age group. Analyses accounted for variations by age, sex, and socio-demographic index (SDI). Joinpoint regression analysis identified years of significant trend shifts.

**Results:**

The global incidence of T2DM among adolescents and young adults rose from 56.02 per 100,000 (95% UI 43.03–72.32) in 1990 to 123.86 per 100,000 (95% UI 100.43–149.79) in 2021, reflecting an AAPC of 3.01 (95% CI 2.78–3.23). Notable increases were recorded in 1995, 2002, and 2009, with joinpoints indicating significant trend stabilization post-2010 for prevalence and DALYs. The largest relative incidence increase was observed in the 15–19 age group [AAPC 2.97 (95% CI 2.71–3.24)]. Although T2DM mortality was 2.4 times higher in the 15–19 age group compared to the 20–24 age group, the latter exhibited a significantly higher overall mortality rate. Regionally, Oceania recorded the highest incidence rates in 2021, while North Africa and the Middle East showed the greatest AAPCs. High-SDI countries experienced the most substantial increase in T2DM burden, with males comprising 54.8% of cases.

**Conclusions:**

From 1990 to 2021, the global burden of T2DM among adolescents and young adults has markedly increased, underscoring the necessity for targeted, region-specific interventions to address this issue. The observed demographic disparities in mortality rates necessitate the implementation of age-specific strategies. Furthermore, the emergent trends in T2DM indicators warrant urgent attention to mitigate the rising burden in this vulnerable population.

**Graphical abstract:**

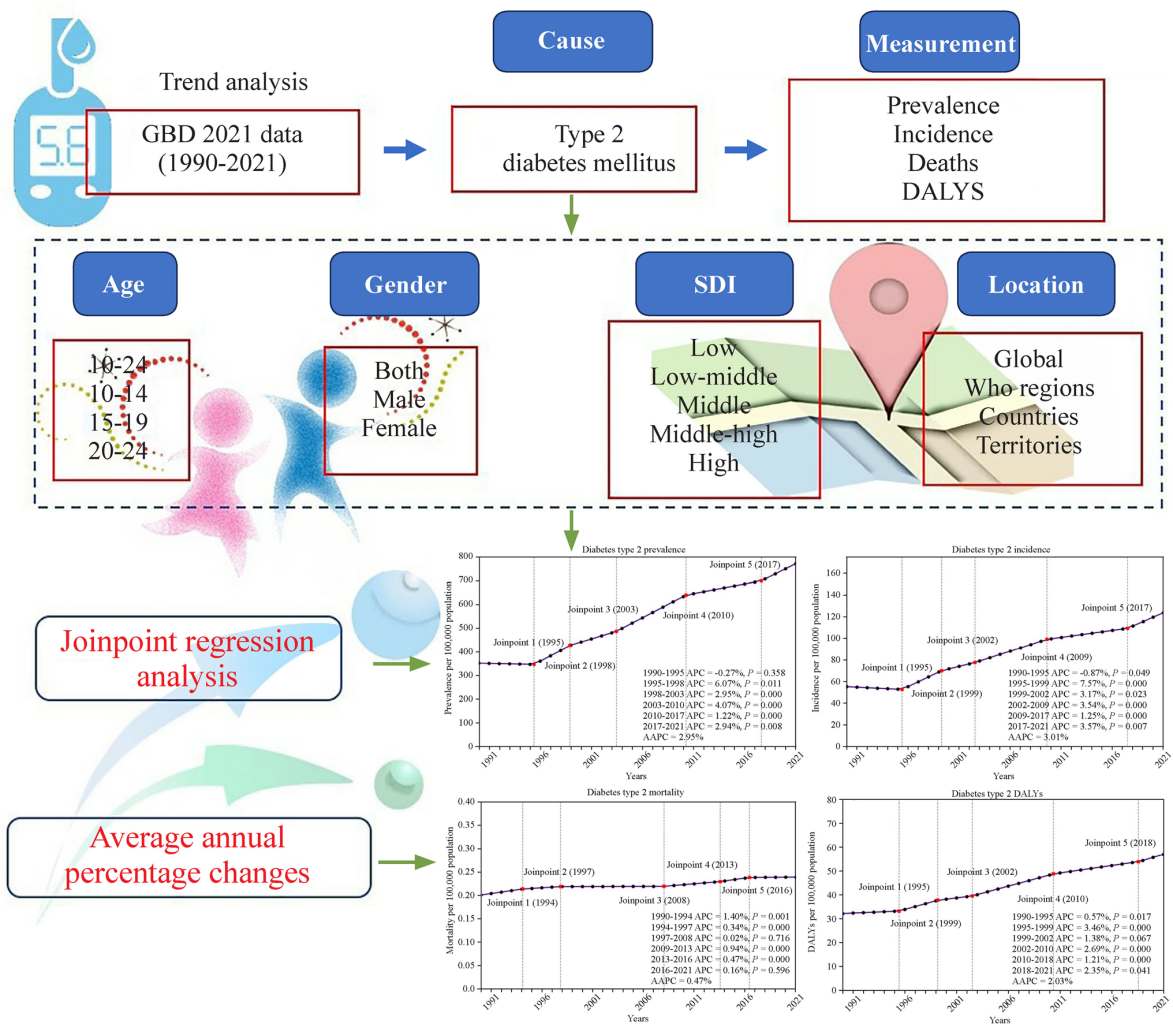

## Introduction

Over 537 million adults aged between 20 and 79 years were estimated to have diabetes in 2021, with the figure expected to rise to 783 million by the year 2045 [1]. Type 2 diabetes mellitus (T2DM) has traditionally been viewed as a condition primarily affecting individuals in middle to late adulthood, with adolescents and younger adults rarely diagnosed. However, early onset T2DM is increasingly becoming established, as evidenced by reports indicating exponential rises in its prevalence among young people. In the decade between 2002 and 2012, the prevalence among this demographic increased by nearly 5% annually [2].

Over the past few decades, both the prevalence and incidence rates of T2DM have increased dramatically among younger populations worldwide [3–5]. This rising trend is influenced by genetic predisposition, lifestyle and behavioral changes, increasing obesity rates, and other environmental factors [6, 7]. Adolescents and young adults are living unhealthy lifestyles due to poor diets and physical inactivity, largely related to urbanization and globalization [8, 9].

Despite the increasing recognition of T2DM as a health problem among young individuals, comprehensive data regarding the burden and trends of the disease in these cohorts remains scarce [10, 11]. Central to this understanding is the fact that these trends signal calls for developing and implementing prevention and management strategies [12, 13]. The Global Burden of Diseases, Injuries, and Risk Factors Study (GBD) 2021 provides valuable insights into the global burden of T2DM, including its incidence, prevalence, and disability-adjusted life years (DALYs) across various regions and population groups [3, 14].

In this study, we describe the global trends for prevalence, incidence, mortality, and DALYs of T2DM by decade since 1990, identifying the years with the most significant rate changes. Moreover, we stratify these trends by age, sex, and social development index (SDI), reporting the results on regional and national levels. The findings from this analysis will help to inform and tailor public health policies and interventions to effectively address the critical issue of T2DM among adolescents and young adults [10, 13].

## Methods

### Study population and data collection

For our analysis of the GBD 2021, we used repeated cross-sectional GBD data from the Global Health Data Exchange (GHDx), which includes the global burden of 372 diseases and injuries in 200 countries and territories from 1990 to 2021, including T2DM [14].

Diabetes mellitus was defined in the GBD 2021 study as a fasting plasma glucose concentration ≥ 126 mg/dL (7 mmol/L) or reported treatment for diabetes [15]. T2DM mortality was estimated from vital registration sources by aggregating the death records based on ICD-10 (International Classification of Diseases, 10th Revision) codes. The crude years lived with disability were computed by multiplying the prevalence of T2DM with the respective disability weights for this health state.

The World Health Organization (WHO) defines adolescence as the age range between 10 to 19 years, characterized by biological growth and social and behavioral changes [16]. For this study, “adolescents” refers to individuals aged 10–19 years, while “young adults” were those aged 20–24 years. Age subgroups, namely, younger adolescents (10–14 years), older adolescents (15–19 years), and young adults (20–24 years), were used to provide more specific information about growth.

The GBD 2021 also estimated the SDI for each country, defined as a composite index of social and economic development. The SDI is based on the total fertility rate for women under 25, the mean years of education for individuals aged 15 years and older, and lag-distributed income per capita. The index ranges from 0 (lowest education and income, highest fertility) to 1 (highest education and income, lowest fertility). Countries are categorized into five groups based on their SDI: lower, low–middle, middle, high–middle, and high SDI.

Incident cases, prevalent cases, deaths, and DALYs were directly extracted from the GBD 2021. All rates are reported per 100,000 population. The 95% uncertainty intervals (UIs) were defined by the 2.5th and 97.5th percentiles of 1000 estimates based on the GBD's algorithm.

Our analysis followed the reporting guidelines of the Guidelines for Accurate and Transparent Health Estimates Reporting for cross-sectional studies.

### Statistical analysis

The main objective of this study was to determine global trends in the incidence, prevalence, mortality, and DALYs incurred from type 2 diabetes mellitus. We computed age-specific rates and their average annual percentage changes (AAPCs) through linear regression, considering each independent variable as a continuous variable while using the rank order log of the dependent variables. The AAPC is a summary measure that provides an overall description of trends over a specified interval and is calculated as a weighted average of the annual percentage changes (APCs). This enables us to describe average APCs over several years with a single value. In deriving the APC, the geometrically weighted average of annual percentage changes was used in regression analysis. The AAPC describes the yearly percentage change as an increase, decrease, or no change. For example, an AAPC of 0.1 indicates an increase of 0.1% per year. Temporal trends were represented by the estimates of AAPCs and their 95% confidence intervals (CIs).

The second main objective was to determine the year in which the most significant transitions in trends occurred for the indicators cited above. Joinpoint regression analysis, a typical method for electronic medical record (EMR) mining, was used to identify the trends between points over time [17–21]. This approach aimed to fit the simplest model to the data by drawing several connecting line segments on a logarithmic scale. These connecting line segments, referred to as joinpoints, begin from the simplest model, which gives zero joinpoints represented by a straight-line fit. Additional joinpoints were tested for using a Monte Carlo permutation method. The final model was chosen using professional judgment and the weighted Bayesian information criterion measure within Joinpoint software.

The third purpose of this study was to stratify global trends by age group, sex, and SDI. The fourth purpose was to report regional and national trends. As noted above, we used the same AAPC method for these analyses. Inferences from the statistical tests were reported together with effect sizes, confident intervals (Cis), rates, UIs, and exact *P* values. All analyses were conducted using Python version 3.9.13.

## Results

### Global trends

**Fig. 1:**
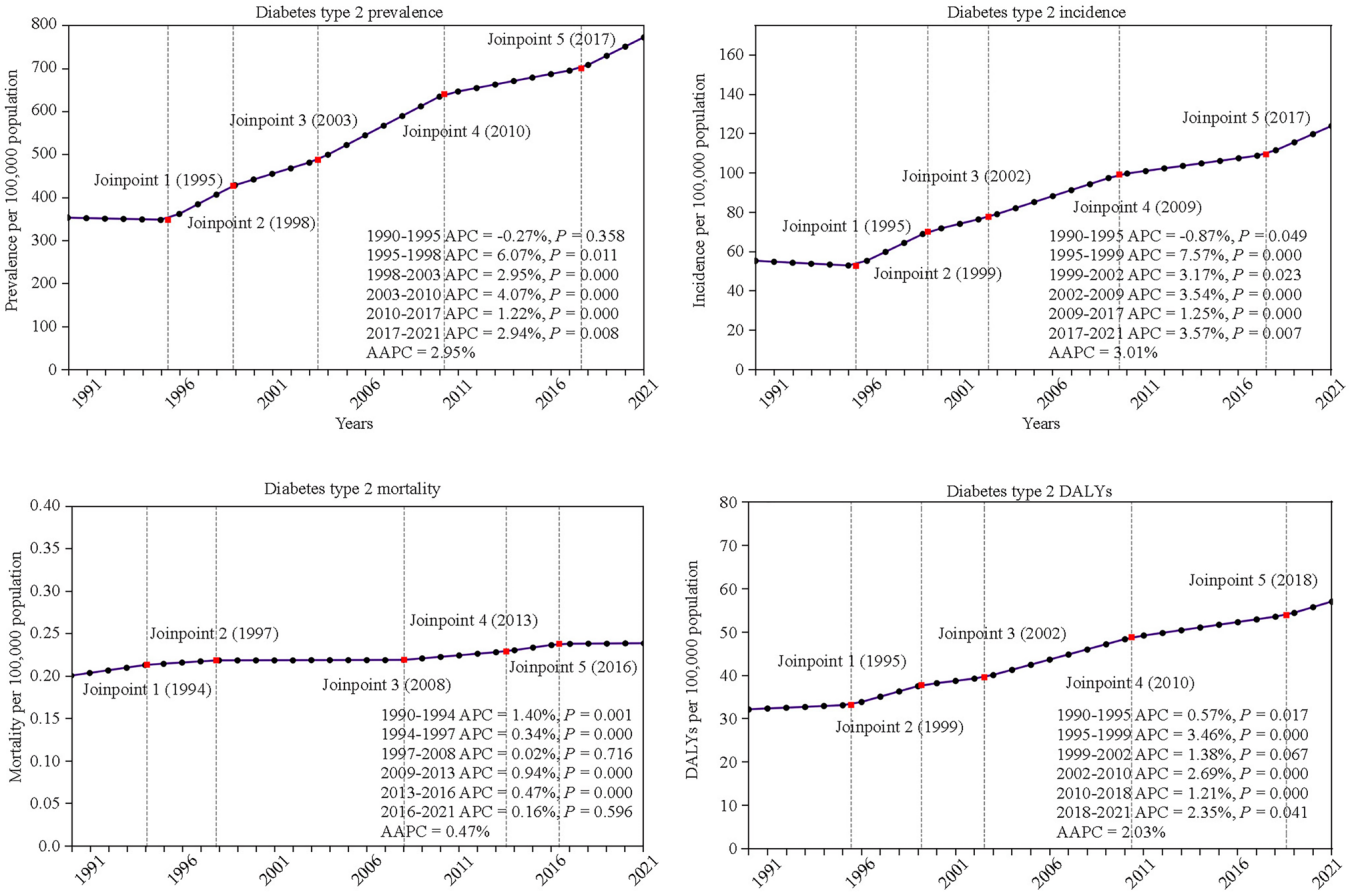
Joinpoint regression analysis of T2DM prevalence, incidence, mortality, and DALYs in adolescents and young adults. *DALY* disability-adjusted life-years; *T2DM* type 2 diabetes mellitus

Between 1990 and 1999, the global incidence of T2DM among adolescents and young adults increased significantly [AAPC 2.03 (95% CI 1.02 to 3.05)], then accelerated further between 2000 and 2009 [AAPC 3.81 (95% CI 3.6 to 4.02)], and continued to rise at a slower rate between 2010 and 2021 [AAPC 1.68 (95% CI 1.47 to 1.89)] (Table 1). In 2021, the incidence of T2DM reached 123.86 per 100,000 population (95% UI 100.43 to 147.79), up from 56.02 per 100,000 population (95% UI 43.03 to 72.32) in 1990, resulting in an overall AAPC of 3.01 (95% CI 2.78 to 3.23). Additionally, T2DM prevalence, mortality, and DALYs increased between 2010 and 2021 (Tables 2 and 3). Joinpoint regression analysis identified significant changes in T2DM incidence trends in 1995, 1999, 2002, 2009, and 2017. Notable joinpoints for prevalence (2010), incidence (2009), and DALYs (2010) indicated phases of significant stabilization in growth rates (Fig. 1).

**Table 1:** Global AAPCs in prevalence, incidence, mortality, and DALYs of T2DM

Age	Years	Prevalence		Incidence		Mortality		**DALYs**	
		AAPC (%, 95%CI)	*P* value	AAPC (%, 95%CI)	*P* value	AAPC (%, 95%CI)	*P* value	AAPC (%, 95%CI)	*P* value
10–24	1990–1999	2.03 (1.02–3.05)	0.004	2.26 (0.87–3.66)	0.012	0.99 (0.82–1.16)	< 0.001	1.62 (1.15–2.1)	< 0.001
	2000–2009	3.81 (3.6–4.02)	< 0.001	3.52 (3.46–3.57)	< 0.001	0.09 (–0.05–0.23)	0.241	2.46 (2.3–2.63)	< 0.001
	2010–2021	1.68 (1.47–1.89)	< 0.001	1.86 (1.56–2.17)	< 0.001	0.7 (0.55–0.85)	< 0.001	1.39 (1.29–1.5)	< 0.001
	1990–2021	2.95 (2.77–3.13)	< 0.001	3.01 (2.78–3.23)	< 0.001	0.47 (0.41–0.53)	< 0.001	2.03 (1.95–2.12)	< 0.001
10–14	1990–2021	NA	NA	NA	NA	NA	NA	NA	NA
15–19	1990–1999	2.18 (1.52–2.85)	< 0.001	2.87 (1.3–4.46)	0.007	1.32 (1.01–1.63)	< 0.001	1.74 (1.61–1.86)	< 0.001
	2000–2009	2.66 (2.54–2.78)	< 0.001	2.64 (2.35–2.94)	< 0.001	–0.12 (–0.38–0.15)	0.427	1.33 (1.22–1.44)	< 0.001
	2010–2021	2.33 (2.18–2.47)	< 0.001	2.42 (2.07–2.76)	< 0.001	0.82 (0.61–1.04)	< 0.001	1.67 (1.62–1.73)	< 0.001
	1990–2021	2.62 (2.52–2.72)	< 0.001	2.97 (2.71–3.24)	< 0.001	0.53 (0.42–0.64)	< 0.001	1.6 (1.57–1.62)	< 0.001
20–24	1990–1999	2.75 (1.49–4.03	0.003	2.62 (1.36–3.91)	0.004	1.55 (1.44–1.67)	< 0.001	2.37 (1.62–3.12)	< 0.001
	2000–2009	2.99 (2.7–3.28)	< 0.001	2.88 (2.52–3.24)	< 0.001	–1.06 (–1.22––0.9)	< 0.001	1.79 (1.6–1.98)	< 0.001
	2010–2021	2.09 (1.85–2.34)	< 0.001	2.15 (1.86–2.44)	< 0.001	1.31 (1.14–1.47)	< 0.001	1.93 (1.77–2.09)	< 0.001
	1990–2021	2.9 (2.74–3.06)	< 0.001	2.84 (2.68–2.99)	< 0.001	0.22 (0.07–0.37)	0.007	2.09 (2–2.18)	< 0.001

*DALYs* disability–adjusted life–year, *AAPC* average annual percentage change, *T2DM* type 2 diabetes mellitus, *NA* not available

**Table 2:** The number and age-standardized rate (per 100 000) of prevalence and incidence of T2DM at the global and regional levels in 1990 and 2021, and its temporal trends from 1990 to 2021

Characteristics	Prevalence								Incidence				
	1990					2021		1990–2021	1990		2021		1990–2021
	Number (95% UI)				ASR (95% UI)	Number (95% UI)	ASR (95% UI)	AAPC (%, 95% CI)	Number (95% UI)	ASR (95% UI)	Number (95% UI)	ASR (95% UI)	AAPC (%, 95% CI)
Overall	5,527,767.89 (4,099,355.80–7248142.21)				357.28 (264.95–468.47)	14,592,799.62 (11,652,572.08–18081928.17)	773.02 (617.27–957.84)	2.95 (2.77–3.13)	866,750.85 (665,796.07–1118873.65)	56.02 (43.03–72.32)	2,338,108.72 (1,895,924.92–2,827,695.35)	123.86 (100.43–149.79)	3.01 (2.78–3.23)
*Sex*													
Male	2,844,231.77 (2,112,947.7–3,702,251.97)				361.82 (268.79–470.97)	7,754,691.45 (6,174,560.12–9,578,802.14)	801.56 (638.23–990.11)	3.08 (2.89–3.27)	460,032.49 (351,600.9–596,770.47)	58.52 (44.73–75.92)	1,281,868.3 (1,041,792.70–1545399.42)	132.5 (107.68–159.74)	3.13 (2.89–3.36)
Female	2,683,536.13 (1,978,372.05–3508830.69)				352.58 (259.93–461.02)	6,838,108.17 (5,451,790.44–8,501,952.36)	743.01 (592.37–923.80)	2.82 (2.64–2.99)	406,718.37 (313,519.23–521,877.85)	53.44 (41.19–68.57)	1,056,240.42 (854,368.01–1288508.45)	114.77 (92.83–140.01)	2.86 (2.65–3.07)
*Socio-demographic index*													
Low	463,914.61 (348,745.73–600,570.44)				298.03 (224.04–385.82)	2,480,775.00 (1,973,909.58–3,053,855.53)	671.54 (534.33–826.67)	2.59 (2.57–2.62)	61,121.27 (47,346.39–78,728.36)	39.27 (30.42–50.58)	340,139.98 (272,472.08–424685.99)	92.07 (73.76–114.96)	2.73 (2.69–2.78)
Low–middle	1,154,299.52 (859,147.42–1,491,042.21)				319.12 (237.52–412.22)	4,203,345.78 (3,312,402.26–5,271,000.88)	760.45 (599.26–953.60)	2.95 (2.87–3.03)	159,973.90 (123,045.87–206,883.70)	44.23 (34.02–57.2)	608,063.00 (489,086.11–763,957.04)	110.01 (88.48–138.21)	3.09 (3–3.18)
Middle	2,448,054.27 (1,831,372.85–3,160,660.32)				446.05 (333.69–575.89)	4,660,095.72 (3,745,768.34–5,725,743.33)	843.08 (677.67–1035.87)	2.57 (2.33–2.81)	379,219.86 (291,068.67–488,465.28)	69.10 (53.03–89.00)	792,580.93 (643,655.39–963,483.28)	143.39 (116.45–174.31)	2.87 (2.55–3.18)
Middle–high	1,088,415.98 (790,610.10–1442350.24)				383.54 (278.60–508.26)	1,945,606.45 (1,531,096.14–2,436,243.72)	861.34 (677.84–1078.56)	3.73 (3.30–4.16)	186,947.84 (140,339.78–243,345.60)	65.88 (49.45–85.75)	381,296.29 (311,291.79–459,212.79)	168.80 (137.81–203.3)	3.58 (3.49–3.67)
High	369,611.57 (235,450.68–522,341.36)				188.71 (120.22–266.69)	1,293,246.55 (999,715.72–1,616,453.92)	696.89 (538.71–871.05)	4.69 (4.53–4.84)	78,942.65 (59,191.29–102,117.30)	40.31 (30.22–52.14)	214,421.81 (171,783.90–263112.46)	115.54 (92.57–141.78)	4.05 (3.58–4.53)
*WHO region*													
African region	452,336.83 (336,764.25–581,834.95)				278.55 (207.38–358.30)	2,249,755.59 (1,790,802.69–2,761,902.22)	597.95 (475.96–734.07)	2.48 (2.39–2.57)	55,972.62 (43,018.70–71951.49)	34.47 (26.49–44.31)	285,711.67 (228,612.50–354734)	75.94 (60.76–94.28)	2.57 (2.48–2.66)
Eastern Mediterranean region	321,252.46 (228,763.23–424,701.60)				267.06 (190.17–353.06)	1,759,132.72 (1,387,798.58–2,200,062.71)	826.08 (651.70–1033.14)	3.91 (3.76–4.07)	49,912.78 (37,810.43–64,392.15)	41.49 (31.43–53.53)	295,415.06 (233,745.14–363,440.68)	138.73 (109.77–170.67)	4.17 (4.05–4.30
European region	310,926.94 (200,401.98–429,970.52)				158.98 (102.47–219.85)	591,650.22 (413,423.4–788,426.46)	361.87 (252.86–482.22)	2.88 (2.75–3.02)	62,209.62 (46,140.34–81,317.99)	31.81 (23.59–41.58)	140,099.46 (110,198.28–174,941.84)	85.69 (67.40–107.00)	3.39 (3.31–3.48)
Region of the Americas	508,037.74 (362,858.43–688,873.56)				256.44 (183.16–347.72)	1,350,920.27 (1,061,495.06–1663762.33)	587.90 (461.95–724.05)	2.76 (2.73–2.78)	79,991.37 (59,969.86–104,364.59)	40.38 (30.27–52.68)	185,201.94 (145,549.73–235,929.47)	80.60 (63.34–102.67)	2.24 (2.22–2.27)
Southeast Asia region	1,375,350.65 (1,021,682.59–1,787,404.22)				343.61 (255.25–446.55)	4,400,565.37 (3,436,031.54–5,550,197.76)	784.86 (612.83–989.90)	2.66 (2.55–2.77)	190,499.63 (147,230.20–246017.22)	47.59 (36.78–61.46)	625,358.94 (493,613.30–792616.27)	111.54 (88.04–141.37)	2.72 (2.58–2.86)
Western Pacific region	2,529,017.62 (1,853,780.03–3310022.21)				546.95 (400.92–715.86)	4,197,785.25 (3,384,369.17–5,075,795.82)	1238.38 (998.41–1497.40)	3.68 (3.26–4.11)	423,205.11 (319,154.73–548,619.69)	91.53 (69.02–118.65)	799,251.70 (655,833.71–956,080.39)	235.79 (193.48–282.05)	4.02 (3.51–4.52)
*Region*													
Central Europe	5466.63 (1449.03–11669.07)				18.72 (4.96–39.96)	1315.13 (6.55–5022.85)	7.25 (0.04–27.70)	− 2.75 (− 3.34– − 2.16)	388,497.41 (291,933.21–504,632.31)	104.38 (78.44–135.59)	725,978.22 (591,773.02–870395.83)	298.74 (243.52–358.17)	1.16 (0.95–1.37)
Eastern Europe	50,545.58 (29,355.15–74,916.06)				107.02 (62.16–158.62)	24,786.15 (10,801.67–43,222.80)	75.12 (32.74–131.00)	− 0.64 (− 1.26– − 0.01)	1908.26 (1072.74–3011.30)	6.54 (3.67–10.31)	1569.99 (756.87–2611.11)	8.66 (4.17–14.40)	1.28 (0.83–1.72)
Western Europe (high income)	179,416.66 (118,070.14–248,032.75)				218.27 (143.64–301.74)	390,331.66 (270,486.31–524,500.92)	541.56 (375.28–727.71)	3.11 (3.02–3.21)	25,475.33 (19,929.80–32001.86)	60.47 (47.31–75.96)	41,122.07 (32,992.90–51157.83)	157.53 (126.39–195.97)	3.3 (3.25–3.35)
Australasia (high income)	1789.57 (672.16–3162.38)				37.20 (13.97–65.73)	5100.54 (2840.31–8656.96)	88.90 (49.51–150.89)	3.98 (2.95–5.02)	2330.30 (1736.25–3002.05)	17.60 (13.12–22.68)	7014.40 (5276.90–9149.51)	45.74 (34.41–59.66)	2.72 (2.47–2.98)
Asia Pacific (high income)	111,315.01 (72,872.11–154,331.02)				264.23 (172.98–366.34)	270,558.82 (210,384.50–338342.65)	1036.43 (805.92–1296.09)	4.57 (4.47–4.67)	40,075.5 (30,188.12–51,875.26)	48.75 (36.72–63.11)	98,113.81 (77,884.61–121,730.48)	136.13 (108.06–168.89)	3.22 (3.12–3.32)
Central Latin America	308,966.25 (241,390.86–387,011.09)				569.47 (444.91–713.31)	620,561.93 (492,969.45–774,799.42)	954.22 (758.03–1191.39)	1.65 (1.60–1.71)	42,521.5 (31,295.44–56,704.7)	28.66 (21.09–38.22)	92,378.50 (70,746.17–117,887.12)	54.02 (41.37–68.94)	1.79 (1.69–1.89)
North America (high income)	41,986.53 (8277.50–90866.17)				68.64 (13.53–148.54)	383,614.76 (295,342.95–480,645.15)	538.19 (414.35–674.31)	1.33 (7.14–7.53)	11,678.96 (6438.93–17,595.76)	19.09 (10.53–28.76)	39,831.92 (29,890.87–51,722.02)	55.88 (41.93–72.56)	3.45 (3.26–3.64)
Southern Latin America (high income)	1649.31 (479.16–3832.02)				12.46 (3.62–28.95)	24,834.68 (13,923.68–38,971.78)	161.93 (90.79–254.11)	9.14 (8.88–9.39)	497.78 (282.14–761.49)	10.35 (5.86–15.83)	1213.80 (747.90–1812.73)	21.16 (13.04–31.60)	3.24 (3.16–3.32)
Andean Latin America	22,918.63 (17,400.62–29,124.30)				186.17 (141.35–236.58)	67,732.69 (53,590.83–83,733.40)	392.33 (310.41–485.01)	2.50 (2.42–2.58)	42,489.15 (33,926.84–53,565.80)	78.31 (62.53–98.73)	89,108.24 (71,000.11–111,347.38)	137.02 (109.18–171.22)	2.71 (2.61–2.82)
Tropical Latin America	93,479.66 (60,136.19–131,091.47)				195.32 (125.65–273.91)	158,070.34 (105,825.21–216,956.44)	312.54 (209.24–428.97)	1.40 (1.32–1.48)	40,246.77 (30,318.17–52,032.82)	36.96 (27.84–47.78)	231,233.41 (183,700.09–282646.51)	142.48 (113.19–174.15)	1.75 (1.68–1.82)
Caribbean	44,333.22 (32,741.00–57756.80)				415.15 (306.6–540.86)	105,942.34 (85,838.75–129,834.28)	935.27 (757.79–1146.19)	2.66 (2.62–2.71)	2670.87 (2008.67–3564.68)	21.70 (16.32–28.96)	8305.17 (6436.51–10,763.61)	48.11 (37.28–62.35)	2.73 (2.69–2.77)
North Africa and Middle East	262,860.26 (187,328.61–348,976.29)				241.36 (172.01–320.44)	1,300,919.15 (1,004,386.20–1608274.35)	801.57 (618.86–990.94)	4.03 (3.89–4.17)	175,437.95 (135,442.84–227,932.73)	52.45 (40.49–68.15)	647,713.70 (511,280.70–823514.5)	123.17 (97.23–156.60)	4.57 (4.46–4.69)
Central Asia	38,277.80 (26,129.88–52,225.57)				193.02 (131.76–263.35)	79,233.58 (57,745.9–103,775.53)	358.08 (260.97–468.99)	2.53 (2.19–2.87)	5568.13 (4218.13–7359.84)	28.08 (21.27–37.11)	14,859.95 (11,709.35–18,699.15)	67.16 (52.92–84.51)	3.32 (3.04–3.59)
South Asia	1,302,552.98 (974,598.91–1,675,912.90)				389.44 (291.39–501.07)	4,605,364.35 (3,587,280.15–5,788,299.54)	875.76 (682.16–1100.71)	2.75 (2.66–2.83)	7748.81 (6088.55–10,001.99)	44.77 (35.18–57.79)	49,251.04 (39,050.77–61,646.53)	109.58 (86.89–137.16)	2.88 (2.78–2.99)
East Asia	2,324,510.43 (1,705,676.45–3,025,443.91)				624.57 (458.29–812.90)	3,709,958.43 (3,000,143.46–4,493,325.83)	1526.66 (1234.57–1849.02)	4.05 (3.60–4.51)	6191.89 (4847.49–7926.55)	57.98 (45.39–74.23)	14,789.24 (11,852.70–18405.25)	130.56 (104.64–162.48)	4.45 (3.91–5.00)
Southeast Asia	289,367.75 (196,197.20–391224.89)				195.04 (132.24–263.69)	582,238.88 (433,434.20–746891.51)	340.47 (253.46–436.76)	0.95 (0.62–1.29)	2755.92 (2248.02–3405.33)	131.74 (107.46–162.79)	13,073.87 (10,907.88–16,020.56)	324.22 (270.51–397.30)	1.29 (0.98–1.61)
Oceania	17,056.74 (13,821.11–20,893.08)				815.37 (660.69–998.75)	73,316.61 (61,216.27–88,160.44)	1818.20 (1518.12–2186.31)	2.50 (2.43–2.58)	10,080.04 (7134.73–13,778.04)	21.34 (15.11–29.17)	9267.82 (6253.78–12,867.76)	28.09 (18.95–39.00)	2.80 (2.71–2.88)
Central sub-Saharan Africa	64,757.60 (51,376.64–81,431.01)				374.15 (296.84–470.49)	372,972.79 (306,188.26–453,443.03)	829.86 (681.27–1008.91)	2.52 (2.46–2.58)	6761.82 (5132.73–8859.13)	39.58 (30.04–51.86)	15,696.08 (12,279.20–19750.27)	71.95 (56.29–90.54)	2.82 (2.76–2.88)
Southern sub-Saharan Africa	51,548.44 (37,303.88–67,300.09)				301.74 (218.36–393.94)	120,648.94 (93,954.82–149,287.50)	553.07 (430.70–684.35)	2.06 (1.84–2.29)	21,940.33 (16,663.02–28511.55)	36.66 (27.84–47.64)	137,861.27 (109,115.43–171,604.57)	85.43 (67.62–106.35)	2.07 (1.86–2.27)
Eastern sub-Saharan Africa	108,461.12 (73,770.52–149,840.27)				174.84 (118.92–241.55)	509,637.49 (383,693.21–652,984.21)	350.42 (263.82–448.98)	2.01 (1.93–2.09)	15,411.11 (11,179.59–20,969.40)	32.2 (23.36–43.81)	27,509.06 (20,083.35–37,084.96)	54.39 (39.71–73.32)	1.87 (1.80–1.93)
Western sub-Saharan Africa	206,507.75 (159,901.71–259,290.51)				345.08 (267.2–433.28)	1,185,660.37 (945,244.88–1,449,521.18)	734.77 (585.78–898.28)	2.55 (2.44–2.66)	16,463.02 (12,607.42–21,064.21)	26.54 (20.32–33.96)	72,217.15 (57,345.76–90,410.68)	49.66 (39.43–62.17)	2.89 (2.78–2.99)

*T2DM* type 2 diabetes mellitus, *UI* uncertainty interval, *ASR* age-standardized rate, *AAPC* average annual percentage change, *CI* confidence interval, *WHO* World Health Organization

**Table 3:** The number and age-standardized rate (per 100 000) of mortality and DALYs of T2DM at the global and regional levels in 1990 and 2021, and its temporal trends from 1990 to 2021

Characteristics	Mortality					DALYs				
	1990		2021		1990–2021	1990		2021		1990–2021
	Number (95% UI)	ASR (95% UI)	Number (95% UI)	ASR (95% UI)	AAPC (%, 95% CI)	Number (95% UI)	ASR (95% UI)	Number (95% UI)	ASR (95% UI)	AAPC (%, 95% CI)
Overall	3119.69 (2715.30–3464.48)	0.20 (0.18–0.22)	4525.87 (3916.90–5210.77)	0.24 (0.21–0.28)	0.47 (0.41–0.53)	503,832.00 (387,014.43–659,434.64)	32.56 (25.01–42.62)	1,077,230.33 (790,659.29–1,439,357.88)	57.06 (41.88–76.25)	2.03 (1.95–2.12)
Sex										
Male	1233.31 (1045.53–1412.33)	0.16 (0.13–0.18)	1998.07 (1654.85–2441.56)	0.21 (0.17–0.25)	0.21 (0.15–0.27)	232,044.18 (171,817.79–309,297.39)	29.52 (21.86–39.35)	540,516.81 (391,640.96–736,210.54)	55.87 (40.48–76.10)	1.74 (1.67–1.81)
Female	1886.38 (1573.49–2140.24)	0.25 (0.21–0.28)	2527.80 (2103.23–2876.23)	0.27 (0.23–0.31)	0.85 (0.79–0.90)	271,787.82 (210,168.18–347,965.89)	35.71 (27.61–45.72)	536,713.52 (397,477.37–713,467.90)	58.32 (43.19–77.52)	2.36 (2.25–2.46)
Socio-demographic index										
Low	750.11 (579.25–893.22)	0.48 (0.37–0.57)	1669.13 (1345.36–2037.16)	0.45 (0.36–0.55)	− 0.48 (− 0.60- − 0.36)	77,372.23 (61,651.13–93,622.38)	49.71 (39.61–60.15)	249,468.36 (192,169.35–320,572.05)	67.53 (52.02–86.78)	0.80 (0.72–0.86)
Low–middle	905.23 (741.21–1052.55)	0.25 (0.20–0.29)	1527.17 (1289.00–1766.98)	0.28 (0.23–0.32)	0.22 (0.14–0.31)	123,405.69 (96,053.78–156,148.47)	34.12 (26.56–43.17)	72,119.02 (45,265.49–104,900.56)	38.86 (24.39–56.53)	1.79 (1.77–1.82)
Middle	131.12 (122.13–142.04)	0.07 (0.06–0.07)	1063.15 (966.33–1179.23)	0.19 (0.17–0.21)	− 0.10 (− 0.23–0.03)	202,667.66 (153,061.00–270894.75)	36.93 (27.89–49.36)	326,898.27 (242,469.46–440,827.32)	59.14 (43.87–79.75)	1.73 (1.60–1.87)
Middle–high	1095.91 (988.45–1202.88)	0.20 (0.18–0.22)	171.85 (154.87–191.17)	0.08 (0.07–0.08)	− 0.17 (− 0.28- − 0.06)	71,737.19 (48,615.31–103,457.22)	25.28 (17.13–36.46)	111,681.31 (71,270.37–162,005.67)	49.44 (31.55–71.72)	3.04 (2.72–3.35)
High	232.95 (196.73–268.51)	0.08 (0.07–0.09)	88.35 (79.89–98.45)	0.05 (0.04–0.05)	− 1.03 (− 1.17- − 0.9)	28,155.12 (19,474.89–40,227.17)	14.38 (9.94–20.54)	316,100.24 (222,132.39–434,330.96)	57.19 (40.19–78.58)	3.59 (3.44–3.73)
WHO region										
African region	885.12 (699.69–1039.75)	0.55 (0.43–0.64)	2027.72 (1616.46–2455.74)	0.54 (0.43–0.65)	− 0.166 (-0.29- − 0.04)	86,635.11 (69,191.6–102,844.5)	53.35 (42.61–63.33)	263,839.21 (207,147.63–334,571.25)	70.12 (55.06–88.92)	0.79 (0.70–0.88)
Eastern Mediterranean region	209.88 (166.81–254.54)	0.17 (0.14–0.21)	577.65 (458.31–694.74)	0.27 (0.22–0.33)	1.60 (1.45–1.75)	159,674.49 (108,600.74–231,198.93)	34.53 (23.49–50.00)	134,240.15 (96,897.50–180801.19)	63.04 (45.50–84.9)	3.02 (2.89–3.15)
European region	115.73 (101.49–129.84)	0.06 (0.05–0.07)	101.66 (91.27–112.82)	0.06 (0.06–0.07)	0.22 (-0.13–0.57)	68,287.28 (57,149.72–83,036.81)	34.47 (28.85–41.91)	37,935.43 (24,264.4–55,223.94)	23.2 (14.84–33.78)	2.17 (2.05–2.29)
Region of the Americas	586.94 (568.67–605.41)	0.30 (0.29–0.31)	989.48 (814.69–1213.16)	0.18 (0.15–0.22)	− 1.39 (− 1.75- − 1.02)	130,298.75 (97,645.60–170191.42)	32.55 (24.40–42.52)	102,791.64 (75,101.72–138,388.52)	44.73 (32.68–60.22)	0.88 (0.66–1.1)
Southeast Asia region	850.88 (656.77–1034.74)	0.21 (0.16–0.26)	446.34 (409.64–487.02)	0.19 (0.18–0.21)	− 0.93 (− 1.08- − 0.77)	24,334.10 (16,781.53–34,670.14)	12.44 (8.58–17.73)	296,200.66 (210,816.95–416,439.62)	52.83 (37.60–74.27)	1.41 (1.35–1.48)
Western Pacific region	454.87 (379.50–545.25)	0.10 (0.08–0.12)	370.70 (325.31–432.08)	0.11 (0.10–0.13)	0.50 (0.35–0.65)	31,803.14 (23,902.90–41043.72)	26.44 (19.87–34.12)	239,032.35 (153,903.91–348,478.19)	70.52 (45.40–102.8)	3.18 (2.84–3.51)
Region										
Central Europe	554.30 (390.99–696.15)	0.17 (0.12–0.21)	902.35 (683.49–1130.08)	0.17 (0.13–0.21)	0.94 (0.61–1.28)	105,665.63 (76,034.00–143012.04)	31.59 (22.73–42.76)	300,659.69 (206,817.94–424,284.14)	57.17 (39.33–80.68)	0.27 (0.06–0.49)
Eastern Europe	14.48 (13.57–15.69)	0.05 (0.05–0.05)	20.64 (19.03–22.53)	0.06 (0.06–0.07)	1.56 (0.47–2.67)	1291.31 (1046.23–1734.42)	4.42 (3.58–5.94)	737.43 (622.96–963.18)	4.07 (3.43–5.31)	0.29 (0.09–0.48)
Western Europe (high income)	44.9 (37.99–53.63)	0.11 (0.09–0.13)	1.03 (0.91–1.18)	0.02 (0.02–0.02)	− 2.98 (− 3.18- − 2.77)	11,381.26 (7194.18–17,443.50)	13.85 (8.75–21.22)	20,720.00 (12,018.86–32,187.14)	28.75 (16.68–44.66)	2.53 (2.44–2.62)
Australasia (high income)	1.03 (0.91–1.15)	0.02 (0.02–0.02)	7.27 (6.04–8.55)	0.03 (0.02–0.03)	− 0.86 (− 1.42- − 0.29)	8926.02 (6290.42–12,544.33)	21.19 (14.93–29.78)	21,326.29 (13,729.72–31,319.26)	29.92 (19.26–43.94)	2.10 (1.68–2.52)
Asia Pacific (high income)	36.36 (34.52–37.97)	0.06 (0.06–0.06)	10.43 (9.41–11.53)	0.07 (0.06–0.08)	− 4.50 (− 4.87- − 4.12)	1056.26 (936.85–1208.01)	7.98 (7.08–9.13)	336.14 (198.94–603.69)	5.86 (3.47–10.52)	3.36 (3.13–3.58)
Central Latin America	16.04 (9.10–22.97)	0.77 (0.44–1.10)	257.99 (175.56–355.25)	0.57 (0.39–0.79)	− 1.33 (− 1.96- − 0.69)	35,876.30 (28,029.24–42,423.13)	57.83 (45.18–68.39)	70,083.46 (55,483.67–88,612.41)	40.98 (32.44–51.82)	0.52 (0.30–0.75)
North America (high income)	30.29 (29.26–31.47)	0.04 (0.04–0.04)	29.97 (28.40–31.65)	0.04 (0.04–0.04)	− 1.49 (− 1.76- − 1.21)	4649.03 (2862.43–7599.62)	7.60 (4.68–12.42)	2008.40 (1305.47–3035.76)	13.10 (8.51–19.79)	4.84 (4.72–4.96)
Southern Latin Ameica (high income)	13.91 (12.65–15.21)	0.11 (0.10–0.11)	10.70 (10.20–11.21)	0.01 (0.01–0.02)	− 1.38 (− 1.54- − 1.22)	165.56 (98.02–269.60)	3.44 (2.04–5.60)	14,551.02 (9084.06–21713.98)	55.74 (34.8–83.18)	1.66 (1.41–1.92)
Andean Latin America	22.50 (19.75–25.66)	0.18 (0.16–0.21)	190.72 (170.24–210.84)	0.29 (0.26–0.32)	− 0.034 (− 0.3–0.23)	2001.25 (1361.18–2636.65)	95.67 (65.07–126.04)	90,561.40 (63,711.53–125,420.67)	55.80 (39.26–77.28)	1.27 (1.18–1.36)
Tropical Latin America	257.45 (188.66–333.89)	0.07 (0.05–0.09)	29.26 (23.53–36.25)	0.17 (0.14–0.21)	− 2.42 (− 2.80- − 2.04)	6538.79 (5205.62–8102.04)	61.23 (48.75–75.87)	14,190.34 (11,378.45–17,533.58)	65.05 (52.16–80.38)	− 0.98 (− 1.27- − 0.7)
Caribbean	105.21 (75.85–139.6)	0.61 (0.44–0.81)	110.1 (94.37–128.33)	0.5 (0.43–0.59)	0.84 (0.7–0.99)	3206.59 (2315.44–4328.51)	16.17 (11.68–21.83)	6578.43 (4955.12–8717.72)	163.14 (122.88–216.19)	1.68 (1.60–1.77)
North Africa and Middle East	303.62 (234.41–365.49)	0.51 (0.39–0.61)	74.52 (53.33–99.02)	0.66 (0.47–0.87)	0.96 (0.79–1.14)	2774.19 (2279.94–3446.10)	22.54 (18.52–27.99)	126,063.04 (95,911.13–163,594.19)	78.12 (59.44–101.38)	3.03 (2.90–3.16)
Central Asia	224.35 (215.49–233.23)	0.47 (0.45–0.49)	292.98 (241.7–350.90)	0.18 (0.15–0.22)	1.56 (0.93–2.20)	3459.02 (2110.70–5309.94)	7.32 (4.47–11.24)	85,463.83 (68,225.20–109290.50)	58.76 (46.91–75.15)	2.10 (1.74–2.46)
South Asia	237.90 (230.01–246.30)	0.44 (0.42–0.45)	812.71 (630.87–1023.87)	0.56 (0.43–0.70)	− 0.03 (− 0.21–0.14)	50,994.79 (40,841.44–62,502.42)	34.37 (27.53–42.13)	46,913.95 (34,502.98–64,162.23)	72.14 (53.05–98.66)	1.94 (1.90–1.98)
East Asia	17.26 (14.16–20.12)	0.09 (0.07–0.10)	9.66 (8.76–10.50)	0.05 (0.05–0.06)	− 0.99 (− 1.14- − 0.85)	33,286.01 (26,825.46–41,586.83)	61.35 (49.44–76.65)	11,236.30 (8630.97–14,886.66)	99.19 (76.19–131.42)	3.59 (3.20–3.99)
Southeast Asia	11.81 (10.34–12.85)	0.03 (0.02–0.03)	558.02 (481.83–654.43)	0.33 (0.28–0.38)	− 0.41 (− 0.53- − 0.29)	135,162.77 (87,763.18–201,424.35)	36.32 (23.58–54.12)	6785.81 (5046.29–8930.72)	30.67 (22.81–40.36)	0.08 (-0.08–0.24)
Oceania	509.72 (406.20–611.81)	0.34 (0.27–0.41)	37.95 (31.14–45.75)	0.17 (0.14–0.21)	0.76 (0.62–0.90)	20,669.88 (18,372.16–23,760.13)	43.19 (38.39–49.65)	2732.46 (1865.39–4012.89)	8.28 (5.65–12.16)	1.65 (1.57–1.73)
Central sub-Saharan Africa	57.44 (47.12–68.15)	0.54 (0.44–0.64)	39.58 (28.37–51.76)	0.98 (0.7–1.28)	− 0.16 (− 0.23- − 0.09)	32,463.41 (25,607.90–39309.74)	54.25 (42.79–65.69)	5610.43 (4157.08–7368.85)	32.50 (24.08–42.68)	0.98 (0.92–1.03)
Southern sub-Saharan Africa	62.55 (55.34–70.67)	0.37 (0.32–0.41)	115.64 (109.61–122.92)	0.23 (0.22–0.24)	1.61 (0.89–2.33)	7141.76 (5891.59–8811.60)	41.8 (34.49–51.58)	38,062.99 (27,957.48–50,738.30)	84.69 (62.21–112.89)	1.81 (1.30–2.32)
Eastern sub-Saharan Africa	424.98 (324.97–510.33)	0.69 (0.52–0.82)	892.34 (629.46–1095.63)	0.55 (0.39–0.68)	− 1.06 (− 1.19- − 0.92)	10,813.57 (8316.79–13,670.97)	62.48 (48.05–78.99)	16,549.72 (12,773.71–22,210.31)	32.72 (25.26–43.91)	− 0.32 (− 0.44- − 0.2)
Western sub-Saharan Africa	173.58 (137.81–206.71)	0.16 (0.13–0.19)	122.02 (100.89–147.45)	0.05 (0.04–0.06)	0.2 (− 0.03–0.42)	26,308.61 (20,041.43–34,436.83)	24.16 (18.4–31.62)	196,059.18 (119,413.73–292,504.35)	80.68 (49.14–120.37)	1.14 (0.98–1.30)

*T2DM* type 2 diabetes mellitus, *DALYs*, *UI* uncertainty interval, *ASR* age-standardized rate, *AAPC* average annual percentage change, *CI* confidence interval, *WHO* World Health Organization

### Global trends by sex

From 1990 to 2021, T2DM incidence increased globally for both males and females. Males showed an AAPC of 3.13 (95% CI 2.89 to 3.36), increasing from 58.52 per 100,000 population (95% UI 44.73 to 75.92) in 1990 to 132.5 per 100,000 population (95% UI 107.68 to 159.74) in 2021. In contrast, females had an AAPC of 2.86 (95% CI 2.65 to 3.07), with rates increasing from 53.44 per 100,000 population (95% UI 41.19 to 68.57) to 114.77 per 100,000 population (95% UI 92.83 to 140.01) over the same period (Tables 2 and 3). Both sexes showed increased prevalence, mortality, and DALYs over the three decades (Table 1). In 2021, 54.8% of the 2,338,108.72 global T2DM incident cases occurred in males (1,281,868.30 cases). Furthermore, the AAPC of mortality among females between 1990 and 2021 was nearly four times that of males.

### Global trends by age groups

The most significant increase in T2DM incidence from 1990 to 2021 was observed in the 15–19 age group [AAPC 2.97 (95% CI 2.71 to 3.24)]. The 20–24 age group also experienced an increase in incidence during this period [AAPC 2.84 (95% CI 2.68 to 2.99)]. Data on T2DM incidence for adolescents aged 10–14 years were unavailable. The 15–19 age group not only had the largest increase in incidence, but also in T2DM mortality [AAPC 2.97 (95% CI 2.71 to 3.24)] between 1990 and 2021. Meanwhile, the 20–24 age group exhibited the highest increase in T2DM prevalence [AAPC 2.9 (95% CI 2.74 to 3.06)] and DALYs [AAPC 2.09 (95% CI 2.0 to 2.18)].

Despite the AAPC for T2DM mortality in the 15–19 age group being more than 2.4 times that of the 20–24 age group from 1990 to 2021, the latter group had a significantly higher AAPC for mortality specifically between 2010 and 2021.

### Global trends by SDI

The highest increase in T2DM incidence by SDI quintile was observed in the high-SDI group, rising from 40.31 per 100,000 population (95% UI 30.22 to 52.14) in 1990 to 115.54 per 100,000 population (95% UI 92.57 to 141.78) in 2021 [AAPC 4.05 (95% CI 3.58 to 4.53)]. All SDI quintiles showed increasing trends in T2DM incidence during this period.

Countries across all SDI quintiles experienced increases in prevalence and DALYs from 1990 to 2021. However, mortality decreased in all quintiles except for low–middle SDI countries during this period. In 2021, middle-high SDI quintile countries had the highest T2DM incidence [168.8 per 100,000 population (95% UI 137.81 to 203.3)], while low-SDI quintile countries reached 54.54% of the T2DM incidence of high-SDI countries [92.07 per 100,000 population (95% UI 73.76 to 114.96)] (Tables 2 and 3).

### Regional trends

Regionally, the largest increases in T2DM incidence between 1990 and 2021 were observed in North Africa and the Middle East, from 52.45 per 100,000 population (95% UI 40.49–68.15) in 1990 to 123.17 per 100,000 population (95% UI 97.23–156.6) in 2021 [AAPC 4.57 (95% CI 4.46–4.69)]; East Asia, from 57.98 per 100,000 population (95% UI 45.39–74.23) to 130.56 per 100,000 population (95% UI 104.64–162.48) [AAPC 4.45 (95% CI 3.91 to 5)]; and North America, from 19.09 per 100,000 population (95% UI 10.53–28.76) to 55.88 per 100,000 population (95% UI 41.93–72.56) [AAPC 3.45 (95% CI 3.26–3.64)] (Tables 2 and 3). In 2021, Southern sub-Saharan Africa had the highest incidence of T2DM at 85.43 per 100,000 population (95% UI 67.62–106.35) (Tables 2 and 3). This region also exhibited the largest increase in T2DM mortality from 1990 to 2021, rising from 0.37 per 100,000 population (95% UI 0.32 to 0.41) to 0.23 per 100,000 population (95% UI 0.22 to 0.24) [AAPC 1.61 (95% CI 0.89 to 2.33)]. At the regional level, high-income regions experienced the largest increases in T2DM prevalence and DALYs between 1990 and 2021. Specifically, Southern Latin America had the largest increase in T2DM prevalence, rising from 12.46 per 100,000 population (95% UI 3.62 to 28.95) to 161.93 per 100,000 population (95% UI 90.79–254.11) [AAPC 9.14 (95% CI 8.88–9.39)]. North America had the most significant rise in DALYs due to T2DM, increasing from 7.6 per 100,000 population (95% UI 4.68–12.42) to 13.1 per 100,000 population (95% UI 8.51 to 19.79) [AAPC 4.84 (95% CI 4.72–4.96)]. In 2021, Central Latin America had the highest prevalence of T2DM, at 954.22 per 100,000 population (95% UI 758.03–191.39); Central sub-Saharan Africa had the highest mortality from T2DM at 0.98 per 100,000 population (95% UI 0.7 to 1.28); and East Asia had the highest DALYs due to T2DM, reaching 99.19 per 100,000 population (95% UI 76.19–131.42).

### National trends

At the national level, Greenland exhibited the most significant increase in T2DM incidence between 1990 and 2021 [AAPC 8.51 (95% CI 8.07–8.95)]. Argentina had the highest rise in T2DM prevalence [AAPC 14.48 (95% CI 13.37–15.6)], while Mauritius saw the most pronounced increases in both T2DM mortality [AAPC 6.47 (95% CI 5.75–7.21)] and DALYs [AAPC 5.94 (95% CI 5.48–6.4)]. In 2021, the country with the highest T2DM incidence was the Marshall Islands at 782.26 per 100,000 population (95% UI 635.86–966.22). China had the highest T2DM prevalence at 1547.76 per 100,000 population (95% UI 1252.46–1877.46). Kyrgyzstan had the highest T2DM mortality at 0.06 per 100,000 population (95% UI 0.05–0.08), and Eswatini had the highest DALYs due to T2DM at 86.84 per 100,000 population (95% UI 59.96–117.53). A global map illustrating the incidence of T2DM in 2021, along with the age-adjusted AAPC in T2DM incidence from 1990 to 2021, categorized by age groups.

## Discussion

This is a comprehensive study to describe the incidence and rates of change of T2DM among adolescents and young adults aged 10–24 years across 200 countries and territories from 1990 to 2021, analyzed at the global, regional, and national levels. A slowing trend in the increase of T2DM incidence among this demographic has been observed since 2009, which may be associated with global efforts in T2DM management. While the overall global incidence of T2DM among adolescents and young adults increased between 1990 and 2021, significant stabilization in the growth rate was observed between 2009 and 2017 (APC 1.25). This rate was about one-third of the increase rate observed from 2002 to 2009 (APC 3.54) and from 2017 to 2021 (APC 3.57). In 2021, male adolescents and young adults accounted for more than half of the incident T2DM cases. The AAPC in mortality from 1990 to 2021 was nearly four times higher in females compared to males. Adolescents aged 15–19 years had an AAPC for T2DM mortality more than 2.4 times that of the 20–24 age group during the same period. However, from 2010 to 2021, young adults aged 20–24 years exhibited a significantly higher AAPC for T2DM mortality. Between 1990 and 2021, the highest increase in T2DM incidence among adolescents and young adults was observed in North Africa and the Middle East. High SDI countries also demonstrated the largest increase in T2DM incidence within this age group.

T2DM is a significant global public health concern, leading countries to implement various strategies and policy guidelines to manage and control the disease. Efforts to combat T2DM include public health campaigns, healthcare infrastructure improvements, and patient education programs. To provide a clearer roadmap for policymakers and public health practitioners, we propose several targeted intervention strategies: (1) implementing school-based and community-wide programs to promote healthy eating and physical activity among children and adolescents; (2) strengthening primary healthcare systems to ensure early diagnosis and continuous management of T2DM, especially in high-risk groups; (3) developing culturally tailored education and awareness campaigns that address specific local risk factors, including dietary habits and sedentary lifestyles; and (4) integrating digital health solutions, such as mobile apps and telemedicine, to improve patient engagement and adherence to treatment plans. These strategies can help guide effective policy implementation and reduce the global burden of T2DM. Based on the GBD data from 2021, the positive percentage contribution of risk factors to DALYs for T2DM among adolescents and young adults aged 10–24 worldwide is ranked as follows: high fasting plasma glucose, metabolic risks, high body-mass index, environmental/occupational risks, suboptimal temperature, high temperature, and low temperature. Among these, high fasting plasma glucose and metabolic risks emerge as the most significant contributors to the burden of T2DM, substantially surpassing other factors. These findings suggest that comprehensive interventions targeting these primary and secondary risk factors are crucial for reducing the burden of T2DM among adolescents and young adults.

Significant stabilization in growth rates was observed at key joinpoints: prevalence in 2010, incidence in 2009, and DALYs in 2010, coinciding with the introduction of global T2DM management strategies. In the USA, the American Diabetes Association (ADA) releases annual guidelines to help healthcare providers in managing diabetes. The 2009 Standards of Medical Care in Diabetes emphasized personalized patient care, the integration of technology, and lifestyle management to improve blood sugar control and reduce complications [22]. In the UK, the National Health Service (NHS) launched the NHS Diabetes Prevention Programme in 2010, aiming to prevent T2DM through lifestyle interventions and support for high-risk individuals. The success of this program has led to its expansion and integration into routine healthcare services [23]. Australia has also made significant strides with the National Diabetes Strategy 2010–2020, focusing on early detection, effective management, and support for research into diabetes prevention and treatment. This strategy underscores the importance of community-based programs and the role of primary healthcare providers [24]. In China, the Ministry of Health released guidelines in 2010 to standardize diabetes prevention and control, including early diagnosis, patient education, and integration of diabetes care into the broader public health system [25]. From 2017 to 2021, there was a renewed moderate increase in T2DM incidence among adolescents, which may be partly attributed to the ongoing obesity epidemic and potential impacts of COVID-19. Rising obesity rates among children and adolescents have significantly influenced T2DM incidence [26]. This partly explains why the AAPC of T2DM mortality in the 15–19 age group was more than 2.4 times that of the 20–24 age group between 1990 and 2021. However, from 2010 to 2021, the AAPC of mortality in the 20–24 age group was significantly higher than that in the 15–19 age group, suggesting a need for targeted T2DM screening among young adults and adolescents. The COVID-19 pandemic appears to be associated with an increase in new-onset T2DM cases in children and higher hospitalization rates in 2020 compared to 2019; however, further studies are needed to differentiate the direct effects of the pandemic from other contributing factors, such as reduced physical activity and changes in diet [27–29]. The pandemic also caused economic and social disruptions, affecting access to healthcare and worsening existing health disparities [30, 31].

From 1990 to 2021, the most significant increase in T2DM incidence among adolescents and young adults occurred in the 15–19 age group. This increase is primarily attributed to hormonal changes during puberty, lifestyle factors such as increased consumption of high-calorie foods, and reduced physical activity. Additionally, socioeconomic disparities affect access to healthcare and healthy food options [32, 33]. The 20–24 age group also experienced a notable rise in T2DM prevalence and DALYs, which may be influenced by factors such as the transition to independent living, educational and occupational stress, and delayed healthcare access. Although the 15–19 age group experienced a higher overall increase in mortality, the 20–24 age group experienced a significant rise in mortality rates between 2010 and 2021. This reflects challenges in managing the transition from pediatric to adult healthcare systems and the chronic nature of diabetes complications [34].

We identified a sex disparity in T2DM among adolescents and young adults. Studies indicate that T2DM incidence and prevalence are higher in adolescent males compared to females [35]. Despite this, females with T2DM exhibit higher DALYs and mortality rates [36]. Notably, our study found female mortality rates to be four times higher than those of males. The increased T2DM incidence in males is attributed to biological and behavioral factors. Hormonal differences, such as the influence of androgens, affect insulin sensitivity and fat distribution, thereby increasing diabetes risk in males [37]. Additionally, adolescent males are more likely to consume sugary drinks and engage in less physical activity than females [38]. Conversely, the higher DALYs and mortality rates in females result from delayed diagnosis, less aggressive treatment, higher susceptibility to complications, and psychosocial factors like depression, which adversely affect diabetes management [36]. Addressing these disparities requires targeted interventions, including early screening and diagnosis, gender-sensitive health education, and comprehensive mental health support for females with T2DM. The gender differences in T2DM underscore the need for gender-specific public health interventions. Understanding these disparities is crucial for developing effective strategies to reduce the T2DM burden.

Regionally, North Africa, the Middle East, East Asia, and North America experienced the most notable rises in T2DM incidence among adolescents between 1990 and 2021. These trends are primarily driven by rapid urbanization, dietary shifts towards high-calorie, processed foods, and reduced physical activity. To address these regional disparities, it is crucial to develop targeted interventions such as improving urban planning to increase access to recreational spaces, providing subsidies for healthier food options in areas with limited access, and launching region-specific campaigns that promote traditional diets and active lifestyles. These strategies should be adapted to the unique socioeconomic and cultural contexts of each region to maximize their effectiveness. Economic development and lifestyle changes, such as increased consumption of western diets and sedentary behavior, further contribute to the increasing incidence. In North America, the obesity epidemic, characterized by high fast-food consumption and low physical activity, plays a crucial role in the rise of T2DM incidence [34]. Socioeconomic disparities also impact access to healthcare and preventive services, exacerbating T2DM management challenges. Southern Sub-Saharan Africa had the highest T2DM incidence rate in 2021, along with a significant increase in mortality. Factors such as limited healthcare access, poor nutrition, and high rates of infectious diseases complicate diabetes management in this region [39]. High-income regions, particularly Southern Latin America, showed the largest increase in T2DM prevalence. North America experienced the largest increase in DALYs due to T2DM, reflecting the chronic nature of diabetes and the burden of related complications. The rapid spread of T2DM among adolescents and young adults in high-SDI quintile countries requires urgent attention. Addressing these regional disparities necessitates targeted interventions, including improved healthcare access, promotion of healthy lifestyles, and effective public health policies tailored to specific regional needs. Nationally, efforts should focus not only on countries with the highest current T2DM burdens, but also on those experiencing rapidly increasing burdens, as unprepared countries are particularly vulnerable to epidemics. Although the widespread T2DM epidemic among adolescents and young adults in lower SDI countries has prompted global and local efforts, the rapid spread in high and middle-high SDI countries also requires urgent action.

The strength of the current study lies in its provision of an up-to-date epidemiological analysis of the global trend of T2DM based on the GBD 2021 findings. This report not only includes the four classic measures—prevalence, incidence, mortality, and DALYs—but also their changing trends at global, regional, and national levels. Additionally, it employs AAPC modeling to estimate the independent effects of age, gender, and region, thereby presenting a clear and multidimensional picture of the T2DM burden over the past three decades.

Our study has several limitations. Firstly, the results are influenced by the methods used in the GBD 2021. In cases of missing data, results relied on predictions from the model, and when data were available, preferred definitions or methods might not have been consistently used, leading to variability, such as in the definition of T2DM across different data sources. This variability may introduce bias in estimating T2DM incidence and mortality rates, potentially leading to either overestimation or underestimation depending on the data source. While techniques were implemented to minimize bias in the GBD 2021, complete elimination of bias is not feasible, meaning the direction of this potential bias could vary, and the magnitude of its impact remains uncertain. Moreover, we used confidence intervals instead of uncertainty intervals after age standardization, which may not fully capture the range of variability in our estimates. This choice could result in a narrower representation of potential outcomes; hence, our findings should be interpreted cautiously, given the possibility of underestimating the true uncertainty. Further real-world studies are needed for validation to better understand the range and impact of these estimates. Secondly, our analysis excluded children and adolescents under 15 years, as the GBD 2021 set 15 years as the age threshold for T2DM. Since T2DM is uncommon in those under 15, we believe that including this age group would not have significantly changed our main findings. Thirdly, the burden of early-onset T2DM depends on detection methods, screening quality, and data registries, all of which vary by socioeconomic level. These variations may lead to systematic biases; for example, countries with more robust healthcare systems may report higher incidence rates due to better detection, while countries with less developed healthcare services might underreport cases, leading to an underestimation of early-onset T2DM in countries with low socio-demographic indices. The direction of this bias is likely toward underestimation in low-resource settings, and the magnitude could be substantial, depending on disparities in healthcare access and data reporting. Fourthly, GBD data may have constraints in economically disadvantaged countries, which often suffer from data scarcity, incompleteness, and potential inaccuracies due to variations in data availability and quality. Despite potentially high T2DM incidence rates in these areas, the sparse data can lead to underestimation or overestimation of the true burden. Consequently, findings based on this data should be interpreted with caution. Fifthly, while our manuscript addresses variations by gender and age, it lacks a more nuanced analysis of specific populations, such as different ethnicities and socioeconomic backgrounds. T2DM incidence and mortality rates in these populations may be influenced by a range of factors, including socioeconomic status, cultural practices, and access to healthcare. We acknowledge that a deeper analysis of these specific subgroups could provide more granular epidemiological insights into the global burden of T2DM. Therefore, future research should aim to perform more detailed analyses of these populations to address this limitation.

In conclusion, the study reveals a marked increase in global incidence and burden of T2DM among adolescents and young adults from 1990 to 2021, with significant regional and demographic disparities. These trends highlight the need for targeted, region-specific interventions, especially in areas with rapid increases. The findings call for urgent policy modifications and early screening programs to address the rising T2DM burden in this vulnerable population.

## Data Availability

The data utilized in this study are derived from the Global Burden of Disease (GBD) database, which is an open-access resource. The datasets generated and/or analyzed during the current study are available in the GBD repository, accessible at Global Burden of Disease Study Data. Researchers interested in replicating or building upon the findings of this study can access the data directly from the GBD repository. Any additional information required to interpret the results reported in this article is available upon reasonable request from the corresponding author.
